# Novel CO_2_-encapsulated Pluronic F127 hydrogel for the treatment of Achilles tendon injury

**DOI:** 10.1038/s41598-023-49339-z

**Published:** 2023-12-11

**Authors:** Yi-Hsun Yu, Chen-Hung Lee, Yung-Heng Hsu, Ying-Chao Chou, Bo-Kui Hong, Chao-Tsai Huang, Shih-Jung Liu

**Affiliations:** 1 Department of Orthopedic Surgery, Bone and Joint Research Center, Chang Gung Memorial Hospital at Linkou, Tao-Yuan, 33305 Taiwan; 2 Division of Cardiology, Department of Internal Medicine, Chang Gung Memorial Hospital-Linkou, Chang Gung University College of Medicine, Tao-Yuan, 33305 Taiwan; 3 Department of Mechanical Engineering, Chang Gung University, Tao-Yuan, 33302 Taiwan; 4 Department of Chemical and Materials Engineering, Tamkang University, New Taipei City, 251301 Taiwan; 5 Biomaterials Lab, Mechanical Engineering, Chang Gung University, 259, Wen-Hwa 1st Road, Kwei-Shan, Tao-Yuan, 33302 Taiwan

**Keywords:** Biological techniques, Medical research, Engineering

## Abstract

Nonsurgical treatment and surgical repairment of injured Achilles tendons seldom restore the wounded tendon to its original elasticity and stiffness. Therefore, we hypothesized that the surgically repaired Achilles tendon can achieve satisfactory regeneration by applying multi-drug encapsulated hydrogels. In this study, a novel bupivacaine-eluting carbon dioxide-encapsulated Pluronic F127 hydrogel (BC-hydrogel) was developed for the treatment of Achilles tendon injuries. The rheological properties of BC-hydrogel were measured. A high-performance liquid chromatography assay was used to assess the release characteristics of bupivacaine in both in vitro and in vivo settings. Furthermore, the effectiveness of BC-hydrogel in treating torn tendons was examined in a rat model, and histological analyses were conducted. Evidently, the degradable hydrogels continuously eluted bupivacaine for more than 14 days. The animal study results revealed that the BC-hydrogel improved the post-surgery mobility of the animals compared with pristine hydrogels. Histological assay results demonstrated a significant reaction to high vascular endothelial growth factor in the surrounding tissues and expression of collagen I within the repaired tendon. This demonstrates the potential of this novel BC-hydrogel as an effective treatment method for Achilles tendon injuries.

## Introduction

A tendon is the anatomical connection between the functional muscle and bone. The tendon itself cannot actively generate a force; instead, it stretches to transmit force from a contracted muscle to the attached bone to create movement. During activities such as walking or running, the Achilles tendon, the largest tendon in the human body, typically experiences significant loads, ranging from 2.63 kN while walking to 3.06–6.64 kN while running^1–4^. A repetitive, high-load, and rapid-transformation force may make a tendon vulnerable. The common patterns of Achilles tendon injury include acute disruption (in active and young adults) and chronic tendinopathy (in older adults). The Achilles tendon is ruptured when the tendon tears partially or completely, causing sudden pain and weakness in the affected leg, which is the most common injury in the middle-aged population who engage in high-impact sports, such as basketball or tennis. This injury can also occur to anyone who participates in physical activity. Treatment of an injured Achilles tendon typically involves a combination of nonsurgical and surgical options^5–7^. Immobilizing the injured leg with a cast or brace is a nonsurgical approach that aims to prevent further harm to the tendon and enable natural healing. Surgical treatment may be necessary in cases where the tear is severe or the nonsurgical treatment is unsuccessful. However, none of these treatments have achieved satisfactory outcomes^8,9^.

Surgical treatment is considered a more suitable method for tissue repair and reinforcement, particularly in active adults, compared to nonsurgical treatments. In addition, researchers have sought adjuvant therapies that facilitate tissue regeneration and guarantee functional performance beyond surgical repairs^10–14^. The conventional therapies focus on improving the bio-availabilities from oral and intravenous routes and topical percutaneous treatment^12–15^. In recent years, implantable pharmaceutical-encapsulated biodegradable polymers have drawn increasing attention, which aims to provide a direct therapeutic treatment at the injured site^16^.

Variable polymers can be incorporated with medications and dissolve completely after performing their therapeutic functions. Polymeric networks, known as hydrogels, can absorb a significant amount of liquid, and the high water content allows hydrogels to mimic the soft and flexible texture of biological tissues^17^. Owing to their similarity to living tissues and excellent biocompatibility, hydrogels have diverse applications in the fields of biomedicine, tissue engineering, and the cosmetic industry, such as wound dressings, contact lenses, and cosmetic products^17–21^. Moreover, biodegradable hydrogels possess huge potential for biomedical applications such as controlled drug delivery^22–24^. Drugs can be loaded into a hydrogel matrix in various ways, including physical entrapment, covalent attachment, or adsorption. The hydrogel can then be administered to patients through various routes, including injection, implantation, and topical application. Following their introduction into the body, hydrogels shield the drug from degradation and gradually dispense it in a regulated manner. The release rate can be modulated by changing the physical and chemical properties of the hydrogel or by incorporating stimuli-responsive components that trigger drug release in response to specific physiological cues. In addition, hydrogels can degrade in vivo and do not need to be removed after a useful lifespan.

The therapeutic benefits of transcutaneous carbon dioxide (CO_2_) treatment have been demonstrated in ischemic tissues, where it enhances local blood flow via the Bohr effect^25,26^. The treatment is now used for peripheral vascular diseases^27^, plastic surgery^28,29^, and chronic wound treatments^30–32^. In addition, CO_2_ therapy has demonstrated its effectiveness in fracture repair^33^, prevention of muscle atrophy after peripheral nerve injury, and improvement of limb contracture after spinal cord injury^34^. Although CO_2_ therapy has been used in several clinical trials, its application has been limited to the transcutaneous pathway. Transcutaneous administration of CO_2_ has the advantage of noninvasiveness; however, the actual percentage of CO_2_ dissolved through the skin is unknown. Previously, a transcutaneous CO_2_ delivery system that utilizes 100% CO_2_ gas, CO_2_ hydrogel, and a CO_2_ adaptor to enhance CO_2_ penetration and ensure a sealed interface with the body surface was developed^26,33^. Nevertheless, this treatment requires a relatively long course, and the bioavailability of CO_2_ remains uncertain.

Moreover, postoperative pain management is crucial because it can help reduce pain and discomfort after surgery and speed up the recovery process. Bupivacaine belongs to the amide group of local anesthetics and is known for its potent analgesic properties^35^. Bupivacaine has been used for regional/epidural/spinal anesthesia and local infiltration by impeding the generation of action potentials in nerve cells, achieved by elevating the threshold for electrical excitement^36^. Previous studies reported that the implantation of bupivacaine-incorporated polymers provided an effective postsurgical analgesic effect and resulted in a faster return to normal activity^37^.

In this study, the local delivery of CO_2_ to the target site was hypothesized to promote the healing of ruptured tendons. A bupivacaine-eluting CO_2_-encapsulated Pluronic F127 hydrogel (BC-hydrogel) was developed, and its efficacy in treating injured Achilles tendons was evaluated. Owing to their controlled drug-release capabilities, hydrogels containing poly(ethylene oxide)-b-poly(propylene oxide)-b-poly(ethylene oxide) (PEO-PPO-PEO), commonly known as Pluronic F127 (PF-127) hydrogels, are frequently used in biomedical applications^38,39^. In the present study, the rheological property of BC-hydrogel was assessed. High-performance liquid chromatography (HPLC) was used to determine the in vitro and in vivo release profiles of bupivacaine. Furthermore, the therapeutic efficacy of the BC-hydrogel in treating ruptured tendons was assessed in vivo using a rat model, along with histological- and mechanical-property evaluations.

## Results

### Characterization of prepared hydrogels

**Figure 1:**
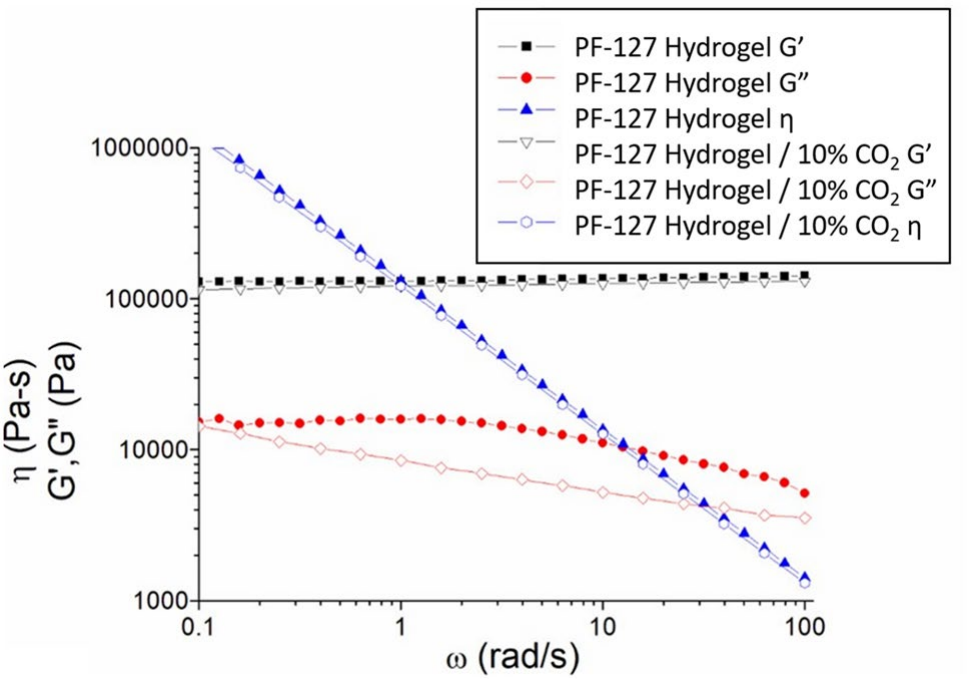
Rheological properties of the pristine hydrogel and 10% CO_2_ incorporated hydrogel.

The rheological properties of the prepared hydrogels were evaluated, as illustrated in Fig. 1. A power-law decrease in viscosity with increasing sweep frequency at 37 °C indicated that both the pristine hydrogel and 10% CO_2_-encapsulated hydrogel (C-hydrogel) exhibited shear-thinning behavior. The 10% C-hydrogel had a lower viscosity compared with the pristine hydrogel. The G′ values of the hydrogels were consistently higher than the G″ values and independent of the sweep frequency. The PF-127 hydrogel exhibited a viscoelastic solid behavior, and its linear viscoelastic response remained constant even at low frequencies. However, with the addition of CO_2_, the composite hydrogels exhibited lower G′ and G″ values. This might result from the plasticizing effect of CO_2_^40^. Additionally, the formation of small bubbles and pores in the composite hydrogels may further reduce both G′ and G″ values. Hydrogels with lower G′ and G″ values are more deformable and can undergo greater strain without significant resistance, facilitating conformity to irregular shapes or deformation with surrounding tissues. Additionally, soft and compliant hydrogels with lower mechanical moduli may better mimic the mechanical properties of natural tissues. This similarity in mechanical behavior can contribute to improved tissue integration, making the hydrogels suitable for tendon healing.

**Figure 2:**
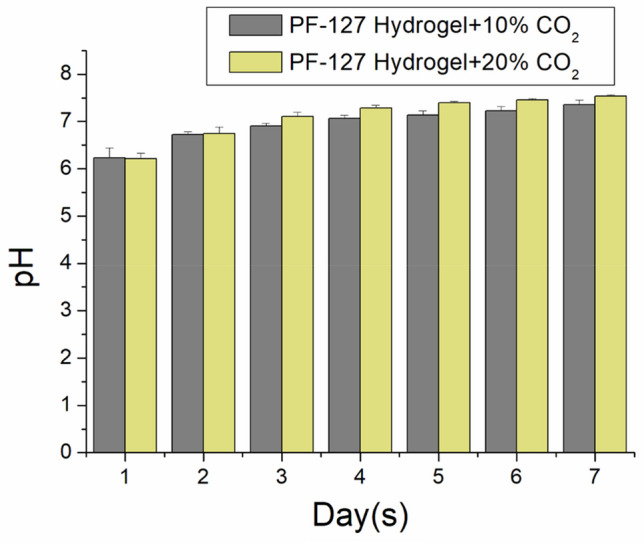
pH-value variation of eluents of CO_2_-encapsulated hydrogels.

Figure 2 illustrates the pH variations of the C-hydrogel, and Table 1 lists the calculated CO_2_ concentrations in the eluents. The pH values of 10% CO_2_ (20% CO_2_) hydrogels increased from 6.23 ± 0.21 (6.21 ± 0.13) on day 1 to 7.36 ± 0.09 (7.54 ± 0.03) on day 7. As CO_2_ interacted with water to form carbonic acid, the hydrogel mixture was initially slightly acidic but gradually became neutral over time. The shift from acidic to neutral conditions may mimic natural physiological changes during tissue healing. This could be relevant in wound healing or tissue engineering, where a hydrogel that adapts to the changing pH conditions of the healing environment could be beneficial.

**Table 1:** Calculated CO_2_ concentrations in the eluents.

Day	10% CO_2_ (M)	20% CO_2_ (M)
1	5.990 × 10^–7^ ± 2.533 × 10^–7^	6.061 × 10^–7^ ± 1.674 × 10^–7^
2	1.604 × 10^–7^ ± 2.939 × 10^–8^	1.583 × 10^–7^ ± 6.196 × 10^–8^
3	9.608 × 10^–8^ ± 1.831 × 10^–8^	1.440 × 10^–7^ ± 1.479 × 10^–7^
4	5.621 × 10^–8^ ± 1.351 × 10^–8^	1.957 × 10^–8^ ± 6.660 × 10^–9^
5	4.368 × 10^–8^ ± 1.566 × 10^–8^	8.250 × 10^–9^ ± 2.491 × 10^–9^
6	2.802 × 10^–8^ ± 1.125 × 10^–8^	3.366 × 10^–9^ ± 2.276 × 10^–9^
7	1.233 × 10^–8^ ± 8.793 × 10^–9^	–2.827 × 10^–10^ ± 5.1 × 10^–9^

**Figure 3:**
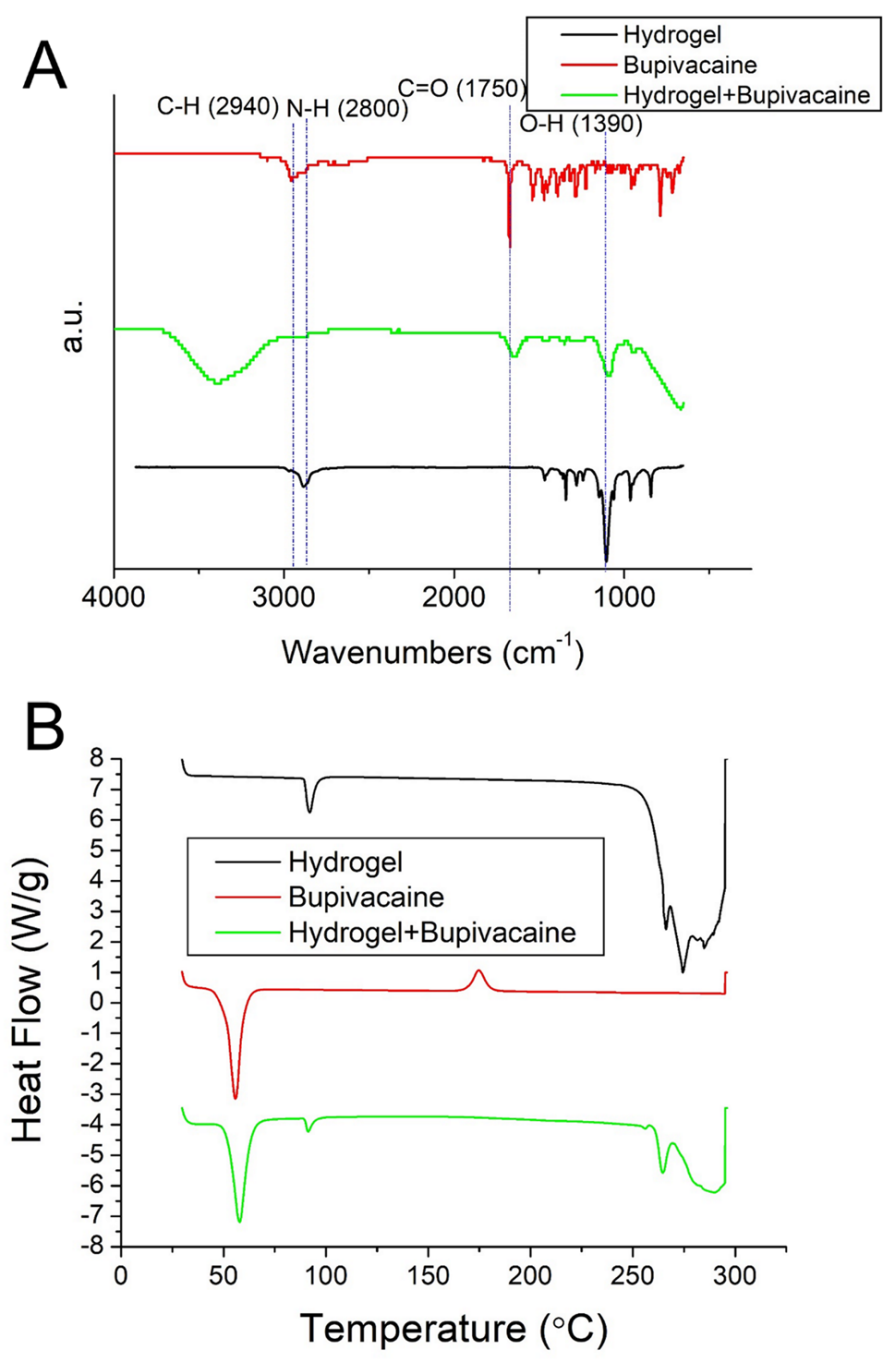
(**A**) FTIR and (**B**) DSC assays of pristine and bupivacaine-eluting Pluronic F127 hydrogels.

Figure 3A illustrates the FTIR spectra of the pristine hydrogel, bupivacaine, and bupivacaine-eluting hydrogel (B-hydrogel). A vibration peak near 1750 cm^–1^, corresponding to the C=O bond of bupivacaine^41,42^, and at 2800 cm^–1^, corresponding to the N–H bond of the drug, could be noted. The peak at 2940 cm^–1^ (C–H bond) was also enhanced by the embedded drug. Figure 3B illustrates the DSC thermograms of the pristine hydrogel, bupivacaine, and B-hydrogel. The exothermal peak of the pristine hydrogel at 55.7 °C and of bupivacaine at 92.2 °C could be identified in the B-hydrogel^43,44^. The results of these analyses indicate that the hydrogels effectively contained the drugs.

### In vitro and in vivo elution of bupivacaine from the hydrogels

**Figure 4:**
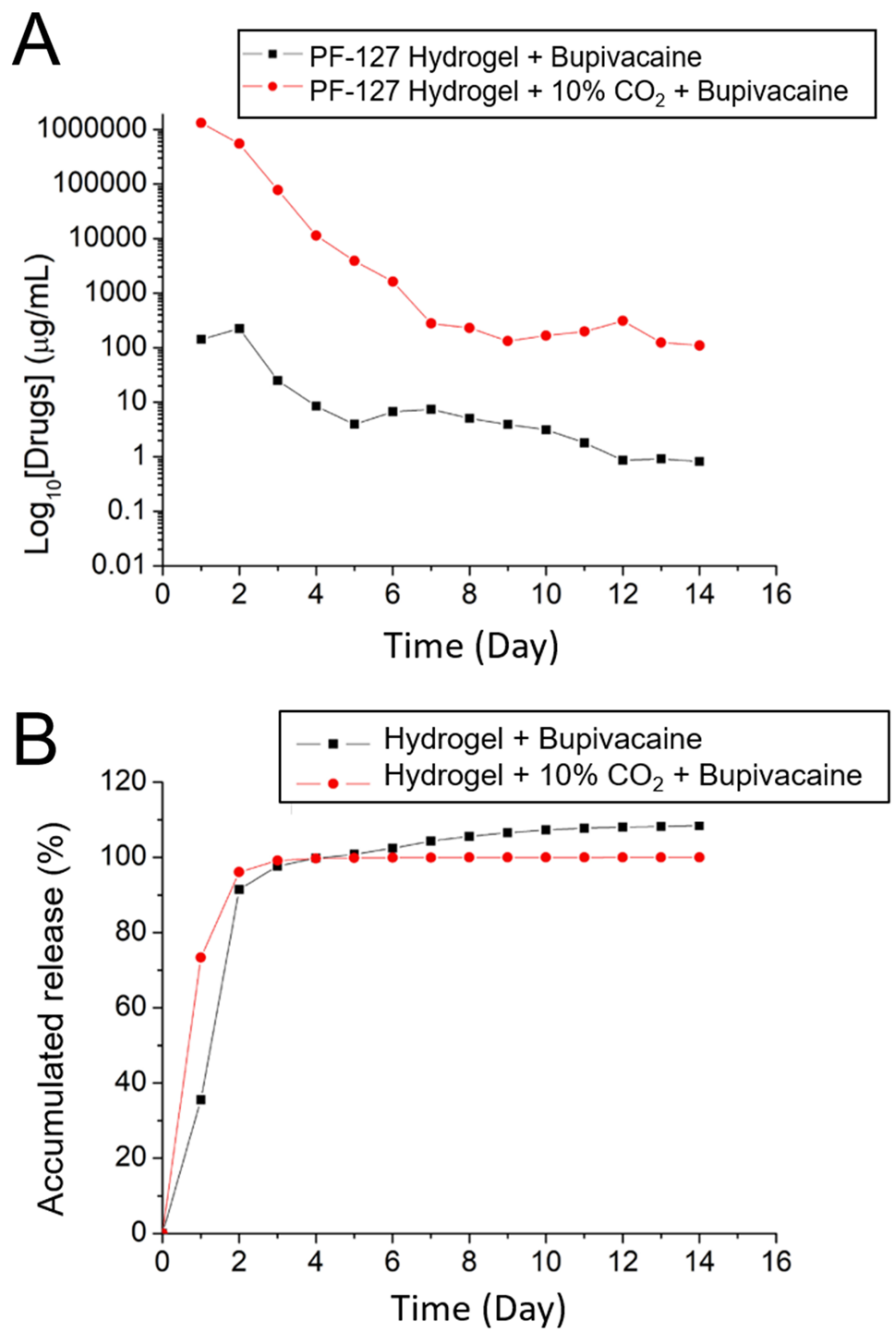
In vitro (**A**) daily and (**B**) accumulated release of bupivacaine from CO_2_-encapsulated hydrogel.

Figure 4A and B show the daily and accumulated in vitro elution profiles of bupivacaine in the B-hydrogel and BC-hydrogel groups, respectively. On days 1 and 2, burst releases were noted, accompanied by a steady and gradually diminishing discharge for up to 14 days. A higher eluted concentration of bupivacaine was observed in the BC-hydrogel group than in the B-hydrogel group.

**Figure 5:**
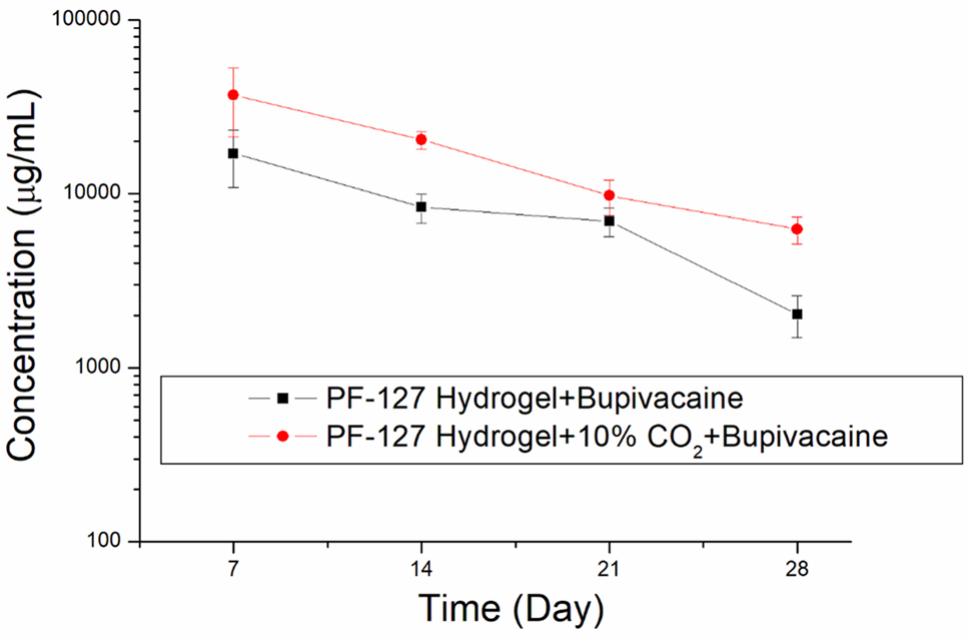
In vivo release of bupivacaine from pristine hydrogel and CO_2_-encapsulated hydrogel.

Figure 5 shows the in vivo elution of bupivacaine from rats in the B-hydrogel and BC-hydrogel groups. The addition of CO_2_ promoted the release of analgesics from PF-127 hydrogels in vivo. Furthermore, the concentrations of both implanted hydrogels remained high until day 28.

### Animals studies

**Figure 6:**
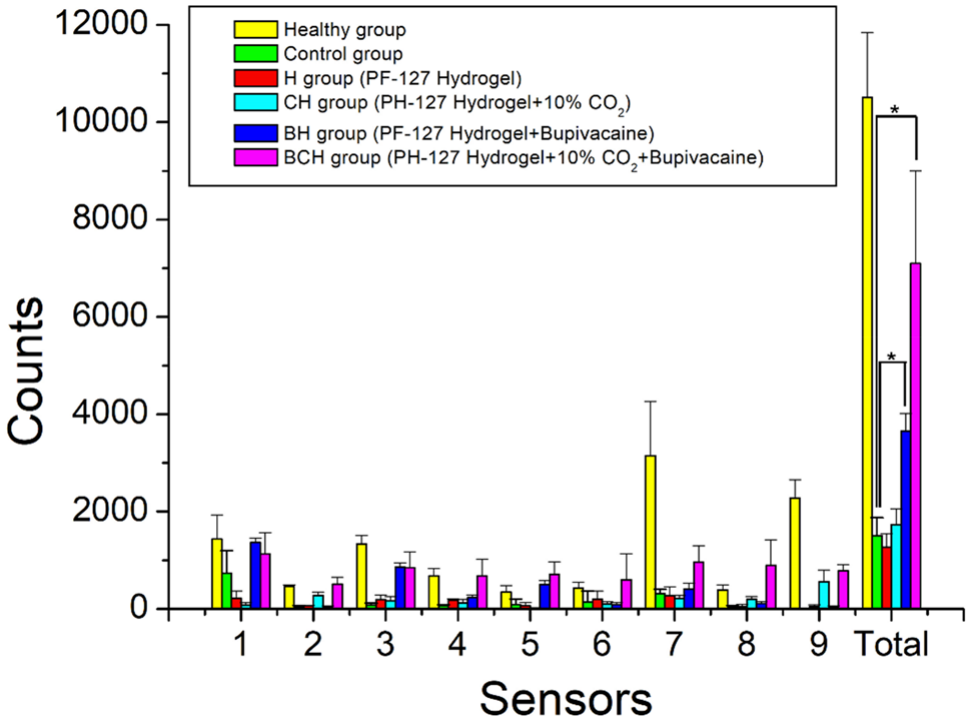
Activity counts of the rats in various groups. *statistical significance.

**Figure 7:**
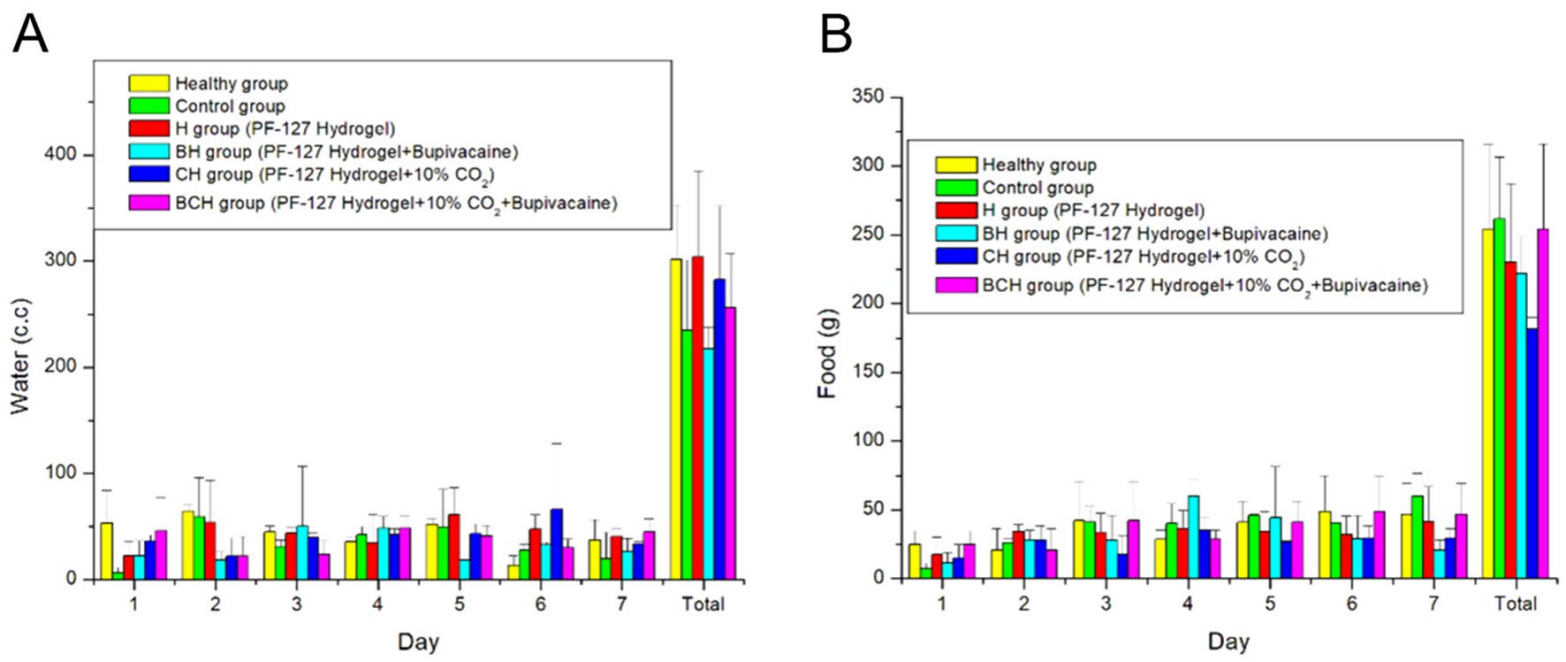
Water and food intake by animals in various groups.

The post-surgical activities of the animals are illustrated in Fig. 6. As expected, the rats in the control group exhibited the lowest activity among the various groups. Rats in the pristine hydrogel and C-hydrogel groups showed activity comparable to that in the control group (P > 0.05). The addition of bupivacaine improved the activity of the rats compared with that of the control (P < 0.05). Finally, the combined effect of bupivacaine and CO_2_ significantly enhanced the recovery of animal activity after the operation relative to the control (P < 0.01), and the rats also exhibited a comparable activity count with healthy animals (P > 0.05). Rats in all groups (Fig. 7) exhibited similar food and water intake levels after surgery (P > 0.05).

**Figure 8:**
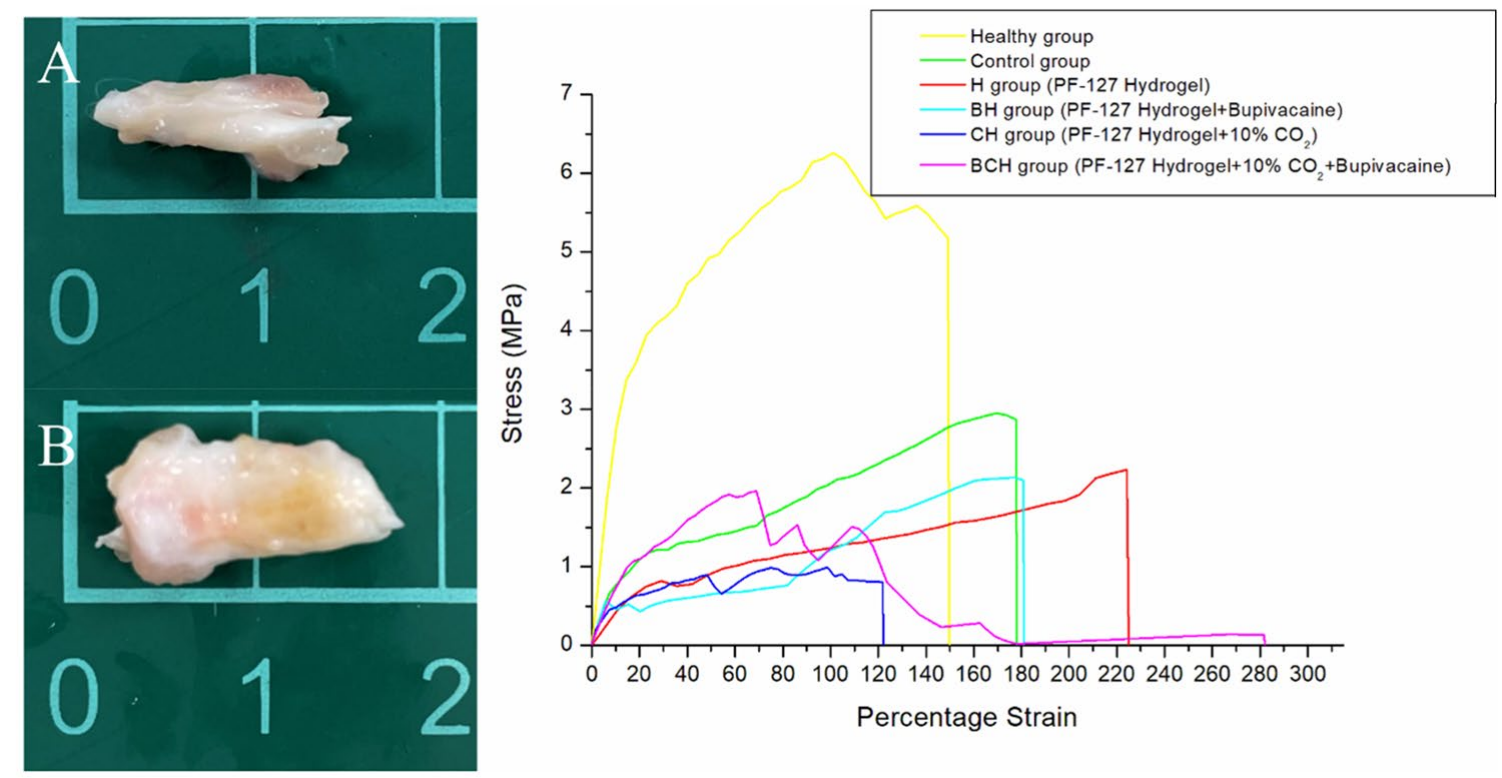
Stress–strain curves of: (**A**) the normal tendon and (**B**) tendons treated with pristine hydrogel and CO_2_-encapsulated hydrogels.

The retrieved Achilles tendons are shown in Fig. 8. When more biomolecules were incorporated into the hydrogel, the cross-sectional area of the specimen increased accordingly (P = 0.03). Additionally, the pristine hydrogel group exhibited inferior tendon strength than the control group, suggesting a possible adverse effect of hydrogels due to their over-aqueous characteristics. The inclusion of CO_2_ was observed to promote the healing of ruptured tendons. Tendons treated with the simultaneous incorporation of CO_2_ and bupivacaine exhibited the best healing outcomes, despite being inferior to those of the control.

**Figure 9:**
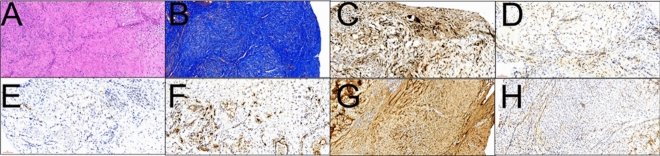
H&E, Masson’s trichrome stain, and IHC examinations of the retrieved tissue and the tendon. (**A**) H&E stain; (**B**) Masson’s trichrome stain; (**C**) VEGF; (**D**) BMP-2; (**E**) TGF-β; (**F**) vWF; (**G**) collagen I; (**H**) collagen III.

Figure 9 illustrates the examination of the muscular tissue and retrieved tendons using various staining techniques and IHC. H&E and Masson's trichrome stains revealed numerous tenocytes distributed throughout well-organized collagen (Fig. 9A and B). Additionally, IHC staining revealed the expression of various growth factors in the cytoplasm, revealing a strong expression of VEGF and moderate expression of BMP-2, TGF-β, and vWF (Fig. 9C–F). Table 2 compares the expression levels of these growth factors. According to the results, the rats treated with CO_2_ exhibited a significant increase in the expression of VEGF compared with the group without CO_2_ (P = 0.03). However, no significant differences were observed in the expression levels of BMP-2, TGF-β, and vWF between the two groups.

**Table 2:** Comparisons of immunohistochemical assays and the corresponding average optical density between the pristine hydrogel and C-hydrogel groups.

	Pristine hydrogel group				C-hydrogel group				p
	IOD	Area	AOD	SD	IOD	Area	AOD	SD	
Muscle									
BMP-2	56,894.66	159,407.00	0.32	0.10	57,988.68	171,068.33	0.34	0.03	0.92
TGF-β	32,977.62	96,746.00	0.34	0.04	38,094.70	124,233.00	0.31	0.02	0.5
vWF	43,371.02	123,208.00	0.35	0.09	71,647.47	218,063.67	0.33	0.10	0.89
VEGF	91,560.01	301,925.67	0.29	0.06	248,556.72	364,675.67	0.69	0.11	0.03*
Tendon									
Masson	462,395.42	1,155,094.67	0.40	0.03	604,866.06	1,301,671.33	0.46	0.03	0.11
Col-I	164,190.54	288,166.33	0.39	0.02	369,426.60	498,765.00	0.74	0.02	0.0002*
Col-III	144,808.28	292,189.00	0.45	0.08	58,319.24	182,322.33	0.32	0.02	0.19

IOD, integrated optical density; AOD, average optical density; SD, standard deviation; BMP-2, bone morphogenic protein-2, TGF-β, tumor growth factor-beta; vWF, von Willebrand factor; VEGF, vascular endothelial growth factor; Col-I, collagen I; Col-III, collagen III.

*Statistical significance.

The retrieved tendons were analyzed for collagen I and III expression levels, as shown in Fig. 9G and H, respectively. Statistically significant collagen I expression was observed in the tendons of the BC-hydrogel group compared with that in the B-hydrogel group (P = 0.0002). However, collagen III expression was statistically similar between the two groups (P = 0.19).

## Discussion

Tendon rupture can cause severe pain and functional impairment, and effective pain management is crucial for the recovery process. Conventional drug administration routes require high doses or frequent administration to elicit a therapeutic response, thus potentially reducing overall effectiveness and patient adherence, and leading to severe side effects or toxicity^6,45^. To address the dilemma, this study developed a novel bupivacaine-eluting CO_2_-encapsulated Pluronic F127 hydrogel for accelerating tendon regeneration after Achilles tendon injury. Based on preliminary results, the therapeutic effects revealed satisfaction with analgesia and enhanced tendon healing in both macroscopic and microscopic evaluations.

Effective and extended drug delivery is essential to improve treatment efficacy. Localized drug-eluting hydrogels allow drugs to effectively diffuse into the surrounding tissues that are difficult to reach by conventional delivery methods^22–24^. Hydrogels consist of a crosslinked polymer network containing a significant amount of water, and the high water content, typically between 70 and 99%, endows the hydrogels with physical characteristics similar to those of tissues. Consequently, hydrogels exhibit excellent biocompatibility and can readily encapsulate hydrophilic drugs^46–48^. Additionally, because hydrogels are typically formed in aqueous solutions, the likelihood of drug denaturation and aggregation owing to exposure to organic solvents, which is required during the fabrication of biodegradable polymers, is minimized. Hydrogel drug delivery systems offer various benefits, including the ability to control the spatial and temporal release of different therapeutic agents, such as small-molecule drugs, macromolecular drugs, and cells^22–24,45–47^. Owing to their adjustable physical properties, manageable degradability, and capacity to shield unstable drugs from degradation, hydrogels can be utilized as a foundation for managing drug release. Through various physicochemical interactions with encapsulated drugs, hydrogels can exert control over their release.

Bupivacaine is a long-acting local anesthetic effective in managing pain after surgery, including tendon-repair surgeries^37,49^. It is a member of the amide family of local anesthetics and has a similar mechanism of action to other drugs in this class. Additionally, it provides targeted pain relief with minimal systemic side effects, thereby reducing the need for additional pain medications^50^. Bupivacaine works by blocking the transmission of nerve impulses in affected areas, thereby reducing pain and inflammation. Moreover, bupivacaine is the superior choice for postoperative pain management because of its long-acting time. Bupivacaine can be administered via various routes, including intravenous, epidural, and local infiltration^51–53^. Currently, bupivacaine is applied using an encapsulated technique to the polymers to guarantee the long-action analgesic effect^37,54,55^. In this study, bupivacaine was co-encapsulated with CO_2_ into the hydrogel with progressive elution to enhance tendon healing under a continuous analgesic condition. Hydrogels facilitate drug release via multiple mechanisms, including diffusion, degradation, and burst release. In the case of bupivacaine embedded within the hydrogel matrix, most of the drug was contained within the hydrogel. However, a small portion may remain on the surface of the hydrogel, resulting in a burst release during the initial 1–2 days. After the initial rapid release, diffusion and degradation mainly controlled the subsequent release of the drug, leading to a gradual and steady decrease in the release of bupivacaine over 14 days. With the addition of CO_2_, small bubbles and pores formed in the composite hydrogels, thereby promoting drug release through channel diffusion. Consequently, the BC-hydrogel group exhibited a higher elution concentration of bupivacaine than the B-hydrogel group. Additionally, because the rate of metabolism in vivo is typically lower than that in vitro, the surrounding environment influences the release mechanism of hydrogels. Consequently, a burst release was not observed in the in vivo curve, and a prolonged release of bupivacaine was noted.

CO_2_ is an essential gas for life and is involved in various physiological processes. CO_2_ therapy is a noninvasive, safe, and effective method for enhancing tissue healing and can be applied in various forms, including inhalation, subcutaneous injections, and topical application^27–34^. It works by increasing blood flow to the target area, which improves the delivery of oxygen and nutrients to the tissues. This increased blood flow further helps remove waste products, such as lactic acid, which can accumulate in damaged tissues and delay healing^56^. In this study, a CO_2_-encapsulated hydrogel was applied to ruptured tendons to enhance blood flow and stimulate tissue regeneration. Encapsulating gas inside the hydrogel for a specific therapeutic goal is a novel endeavor. The CO_2_ can be metabolized through normal physiological reactions without increasing the load on the kidney compared with other encapsulated drugs. Additionally, the hydrogel can be completely dissolved after implantation owing to its high hydrophilicity. Despite the clinical improvements in the post-surgical performances of repaired tendons being insignificant, possibly because the CO_2_ percentage in the hydrogel has been low, increased expressions of VEGF and the content of collagen I were observed. Furthermore, the inclusion of CO_2_ into the hydrogel led to the formation of small bubbles and pores. This may provide an easier pathway for the co-encapsulated drugs to diffuse through these bubbles/pores. Therefore, CO_2_-encapsulated hydrogels exhibited a higher concentration of drug release compared with the pristine hydrogels. The combined effect of bupivacaine and CO_2_ significantly enhanced the recovery of animal activity after the operation relative to the control.

In summary, this study successfully developed a novel bupivacaine-eluting CO_2_-encapsulated Pluronic F127 hydrogel for treating Achilles tendon injuries. Experimental data revealed that the degradable hydrogels achieved a sustained release of bupivacaine for over 14 days in vitro and 28 days in vivo. Moreover, animal tests revealed that CO_2_-encapsulated hydrogels were more effective in promoting post-surgical activity than pristine hydrogels. Histological analysis also indicated a higher expression of VEGF in the surrounding tissues and collagen I within the target tendon. These results highlight the potential of this novel CO_2_-encapsulated drug-eluting Pluronic F127 hydrogel as a promising treatment method for Achilles tendon injuries.

Preliminary work conducted on hydrogels has made significant contributions to the field of tendon healing; however, several limitations need to be addressed. First, the use of pristine hydrogels did not effectively promote the healing of ruptured tendons. This may be owing to the high water content of the hydrogels, which may have hindered the healing process. Therefore, further investigation and optimization of the water content in hydrogels are necessary to improve their effectiveness in promoting tendon healing. Second, the hydrogel vehicles delivered insufficient amounts of CO_2_. This suggests that the delivery system for CO_2_ must be improved to achieve highly effective results. Further research is required to identify the optimal delivery method and dosage of CO_2_ for tendon healing. Despite encouraging results, the applicability of the study's findings to the treatment of tendon ruptures in humans remains uncertain. Therefore, further investigation is needed to determine whether the results can be replicated in humans and whether the treatment is safe and effective for human patients. Future studies should focus on addressing these limitations and advancing the understanding of the potential use of hydrogels in tendon healing.

## Methods

### Preparation of CO_2_-encapsulated drug-eluting hydrogels

**Figure 10:**
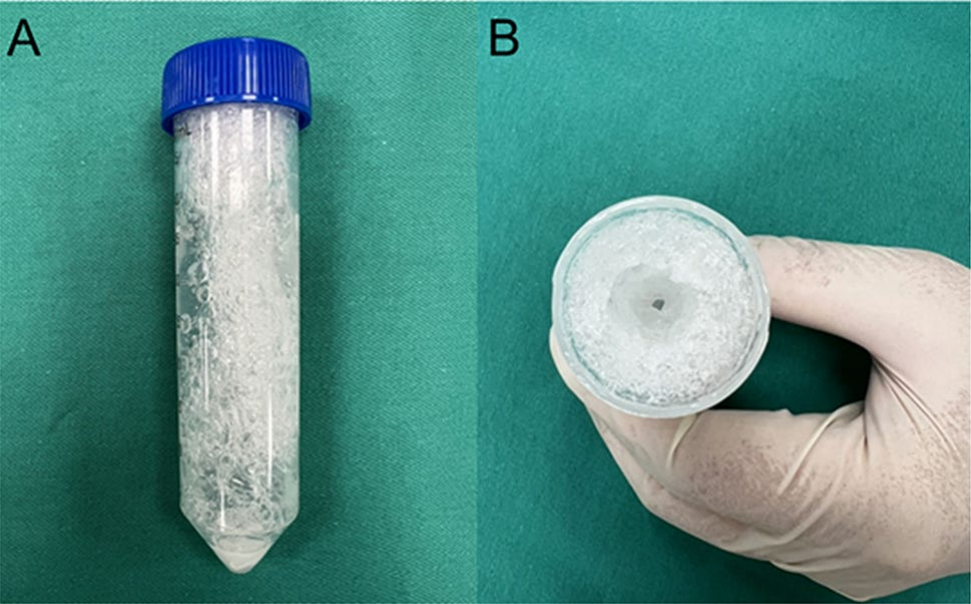
Photographs of the CO_2_-encapsulated hydrogels.

PF-127 hydrogel was obtained from Sigma-Aldrich (St. Louis, MO, U.S.A.). To prepare a pure hydrogel, 5 g PF-127 was dissolved in 10 mL distilled water, and the mixture was stirred for 7 days using a magnetic stirrer. To prepare the 10% CO_2_ hydrogels, 10 g pristine hydrogel was mixed with 1 g dry ice, whereas for the preparation of 20% CO_2_ hydrogels, it was mixed with 2 g dry ice. Figure 10 illustrates the prepared CO_2_-encapsulated PF-127 hydrogel (C-hydrogel).

To prepare the drug-eluting hydrogel, bupivacaine (250 mg) was mixed with pristine hydrogel (3 g; B-hydrogel). To fabricate the BC-hydrogel, bupivacaine (250 mg), pristine hydrogel (3 g), and dry ice (0.325 g) were mixed using a magnetic stirrer for 30 min.

### Rheological properties

A Physica MCR 101 rheometer (Anton Paar, Graz, Austria) was used to assess the rheological properties of pure and 10% C-hydrogels. Frequency sweep tests were conducted at 10 °C with a frequency range of 0.1–100 rad/s and strain of 0.1%. The hydrogel samples were placed on the lower plate of the rheometer and allowed to sit undisturbed for 20 min to ensure thermal stability before testing under isothermal conditions.

### pH value variation

Analysis tubes (n = 3) were filled with 1 mL phosphate-buffered saline (PBS) at a concentration of 0.15 mol/L and pH of 7.4 (Sigma-Aldrich, Saint Louis, MO, U.S.A.) before adding the C-hydrogels. The tubes were kept at a constant temperature of 37 °C for 24 h, and the pH value of the resulting eluent was monitored daily using a pH meter (Starter 3100 PH Bench, Ohaus Corp., Parsippany, NJ, U.S.A.) for 7 days.

### Fourier transform infrared assay

Fourier transform infrared (FTIR) spectrometry was used to analyze the spectra of the pristine and B-hydrogels. The FTIR assay was performed using a Nicolet iS5 spectrometer (Thermo Fisher Scientific, Waltham, MA, U.S.A.). Before the assay, the hydrogel mixture was dried in a vacuum oven at 40 °C for 72 h and compressed into KBr discs. A range of 400–4000 cm^–1^ was scanned at a resolution of 4 cm^–1^, and 32 scans were conducted to obtain the spectra.

### Differential scanning calorimetry (DSC)

To evaluate the thermal properties of both the pure hydrogel and the B-hydrogel, DSC (DSC25, TA Instruments, New Castle, DE, U.S.A.) was used. The specimen was subjected to a heating rate of 10 °C/min over a scan temperature range of 30–250 °C.

### In vitro elution of the pharmaceutical

An in vitro elution method was employed to investigate the release pattern of bupivacaine from the hydrogels. Analysis tubes (n = 3) were filled with hydrogel/bupivacaine mixtures and 1 mL PBS at a concentration of 0.15 mol/L and pH of 7.4 (Sigma-Aldrich, Saint Louis, MO, U.S.A.). The tubes were kept at a constant temperature of 37 °C for 24 h, and the medium in each tube was sampled and replaced with fresh PBS (1 mL) every 24 h over a period of 30 days.

An HPLC system (Hitachi L-2200R, Tokyo, Japan) was used to determine the drug levels in the collected media. A Mightysil RP-18GP (150 × 4.6 mm, 5 µm) B-4 column was used for the assay. The mobile phase comprised a mixture of acetonitrile, distilled water, and orthophosphoric acid in a ratio of 70:30:0.1 (v/v/v). The wavelength and flow rate were set at 210 nm and 2.5 mL/min, respectively. The retention time was set to 2.5 min, and all tests were performed in triplicate (n = 3).

### Surgical procedure

To test the bioavailability and therapeutic capacity of the designed drug-encapsulated hydrogel, Sprague Dawley (SD) male rats weighing 250 ± 20 g on average were used for an Achilles tendon injury simulation animal study. The Institutional Animal Care and Use Committee of Chang Gung University approved the animal experiments (IACUC Approval No.: CGU108-120), and all animal handling procedures adhered to the guidelines and regulations set forth by the Ministry of Health and Welfare of Taiwan. Additionally, the study was conducted in compliance with the ARRIVE guidelines.

Prior to the initiation of surgical procedures, the experimental animals were subjected to a pre-oxygenation period lasting 5 min. Subsequently, isoflurane was administered via a vaporizer (©Matrix, USA) inside a transparent acrylic box (40 cm × 20 cm × 28 cm) to achieve complete anesthesia. Isoflurane was maintained throughout the surgical process to ensure that the animals remained anesthetized and did not experience discomfort or pain.

**Figure 11:**
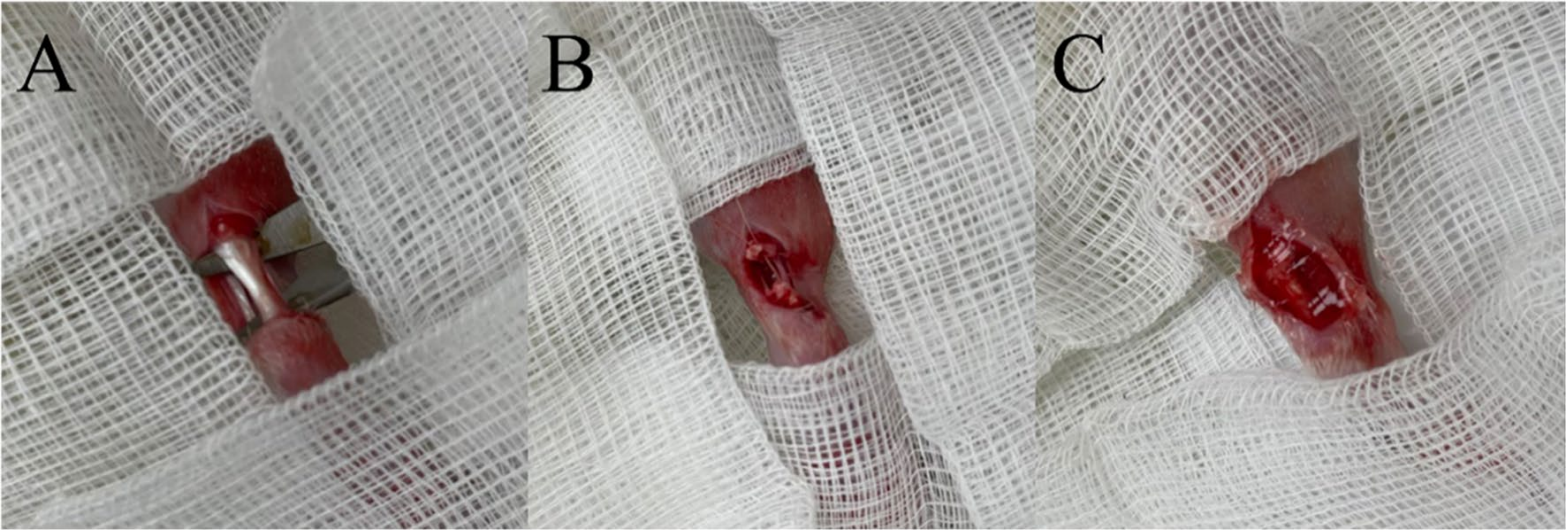
Surgical procedures: (**A**) Achilles tendon is fully identified. (**B**) Mid-portion of the tendon is transected and sutured with absorbable sutures. (**C**) After suturing the tendon, the designed hydrogels are applied around the examined tendon.

The right leg of each animal was then subjected to a standard sterile procedure involving shaving and preparing the area for surgery to minimize the risk of infection and ensure aseptic conditions. Subsequently, a 3 cm-long longitudinal incision was made laterally to the Achilles tendon, and special care was taken to achieve hemostasis. A sharp dissection with a scalpel was then employed to expose the Achilles tendon from the proximally (tendon–muscle junction) to the distally (tendon insertion into the calcaneus) (Fig. 11A). Subsequently, the midportion of the tendon was transected. Using a 5-0 Vicryl suture, the tendon was then repaired end-to-end. (Fig. 11B) Once the transection-repair procedures were completed, the animals were divided into three groups: a normal group consisting of three animals, a control group consisting of three animals, and a study group consisting of 12 animals, all of which were chosen randomly. In the study group, the animals were equally divided into four groups: the pristine hydrogel group, the bupivacaine-eluting hydrogel (B-hydrogel) group, the CO_2_-encapsulated hydrogel (C-hydrogel) group, and the bupivacaine-CO_2_-encapsulated hydrogel (BC-hydrogel) group.

The rats assigned to the normal group were not subjected to any surgical intervention, whereas those in the control group underwent surgery to repair their Achilles tendons without any additional administration of hydrogels. By contrast, the rats assigned to the study group underwent surgery to repair their Achilles tendons, followed by the application of designed hydrogels (average weight, 0.14 ± 0.02 g) around the repaired tendons (Fig. 11C). Subsequently, the surgical incision was sutured using a 3-0 nylon suture, and an antiseptic ointment was applied topically to the wound. The rats were then transferred to their individual cages after regaining consciousness from anesthesia.

### Bioactivities observation

**Figure 12:**
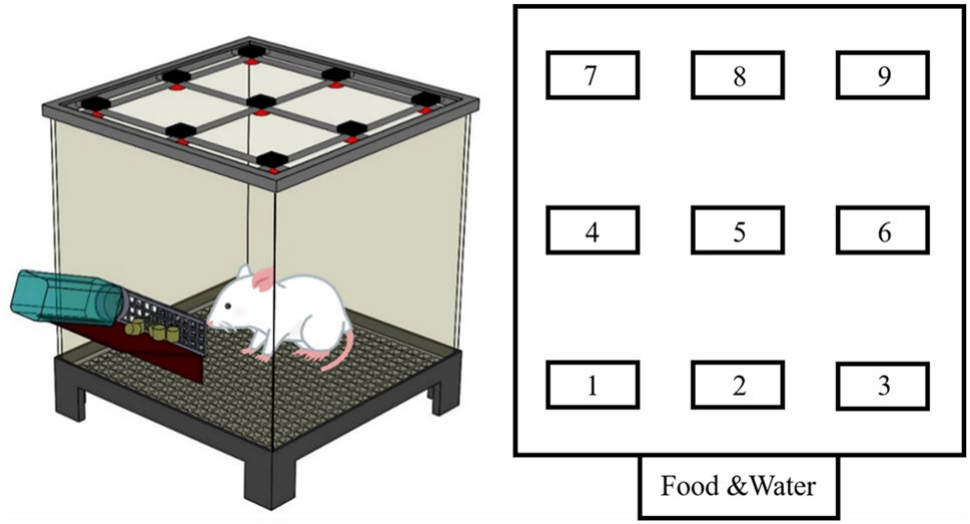
Animal bioactivity cage equipped with movement sensors.

To monitor the rats' postsurgical activity levels and food and water intake, they were placed in a custom-designed animal behavior cage (measuring 50 cm × 50 cm × 50 cm) for 7 days. The animals were monitored daily. To measure movement, the cage was divided into nine cells of equal size, each equipped with a photoelectric switch sensor (HP100-A1; Azbil Corp., Tokyo, Japan) on the top. (Fig. 12) These sensors were spaced 16.7 cm apart for comprehensive monitoring. Whenever a rat moved into a cell, the corresponding sensor triggered and spontaneously recorded its movement. The data were transmitted to a computer via an acquisition interface for further analysis. Daily food and water consumption of the rats was recorded, and the study was performed in a regulated environment with a constant temperature (23–25 °C), pressure (1 atm), and humidity (60–70%). Following the 7 days observation period, the rats were returned to their respective cages.

### In vivo elution of bupivacaine

Muscular tissues (1 × 1 × 1 mm^3^) surrounding the Achilles tendon (B-hydrogel and BC-hydrogel groups) were collected as test specimens using the same anesthesia and surgical dissection methods. Specimens were collected weekly until the experimental rats were euthanized. The collected specimens were preserved in a 4% formaldehyde solution for 24 h before being subjected to HPLC analysis.

### Specimen evaluations

After euthanizing the rats, the experimental Achilles tendons were dissected from the muscular–tendon junction to the point of insertion on the calcaneus. The cross-sectional diameter of the tendon was measured and recorded using digital calipers. A Lloyd tensiometer (AMETEK, USA) was used to evaluate the mechanical properties of both the healthy and experimental tendons. The tendons were stretched at 60 mm/min using a 2.5 kN load cell, and the resulting load–extension curves were recorded.

### Microscopic observations

The extracted Achilles tendons were preserved in 4% phosphate-buffered formaldehyde at room temperature (25–28 °C). Prior to microscopic analysis, tendons were sliced into 2 mm-thick fragments and embedded in paraffin. Tissue sections with a thickness of 4 μm were prepared using a microtome (Sakura Finetek, Tokyo, Japan) and subjected to microscopic assessments using hematoxylin and eosin (H&E) for fundamental compositions and Masson’s trichrome stain for quantification of collagens and tenocytes.

Standard immunohistochemistry (IHC) staining was used to determine the expression of various growth factors. The examined growth factors included vascular endothelial growth factor (VEGF), von Willebrand factor (vWF), bone morphogenetic protein (BMP2), transforming growth factor beta (TGF-β), as well as type I and type III collagens. Commercial antibodies were used for IHC analyses, including VEGF (polyclonal, 1:100, A0280, ABclonal, MA, USA); vWF (vWF Picoband™ antibody, 1:200, PB9062, Boster Biological Technology, Pleasanton CA, USA); BMP2 (polyclonal, 1:50, A0231, ABclonal, MA, USA); TGF-β (polyclonal, 1:50, A2561, ABclonal, MA, USA); type I collagen (1:2000, A1352, ABclonal, MA, USA), and collagen III (1:400, A00788-3, ABclonal, MA, USA). To quantify the growth factors detected in the specimens, the ImageJ software (National Institutes of Health, Bethesda, MD, USA) was used to calculate the average optical density (AOD).

### Statistical analyses

The data were statistically analyzed using SPSS software (version 12.0; SPSS Inc., Chicago, IL, USA) to detect significant differences among the groups. Paired t-tests were used, and a p-value of less than 0.05 was considered statistically significant.

## Data Availability

All data generated or analyzed during this study are included in this published article.
